# Enhancing Equitable Access to Rare Disease Diagnosis and Treatment around the World: A Review of Evidence, Policies, and Challenges

**DOI:** 10.3390/ijerph20064732

**Published:** 2023-03-08

**Authors:** Takeya Adachi, Ayman W. El-Hattab, Ritu Jain, Katya A. Nogales Crespo, Camila I. Quirland Lazo, Maurizio Scarpa, Marshall Summar, Duangrurdee Wattanasirichaigoon

**Affiliations:** 1 Department of Dermatology, Keio University School of Medicine, Tokyo 160-8582, Japan; 2 Department of Medical Regulatory Science, Graduate School of Medical Science, Kyoto Prefectural University of Medicine, Kyoto 602-8566, Japan; 3 United Japanese-Researchers Around-the-World (UJA), Isehara 259-1143, Japan; 4 Department of Clinical Sciences, College of Medicine, University of Sharjah, Sharjah 27272, United Arab Emirates; 5 MENA (Middle East and North Africa) Organization for Rare Diseases, Dubai 500767, United Arab Emirates; 6 Department of Pediatrics, University Hospital Sharjah, Sharjah 72772, United Arab Emirates; 7 Dystrophic Epidermolysis Bullosa Research Association (DEBRA), Singapore 059811, Singapore; 8 Asia Pacific Alliance of Rare Disease Organizations (APARDO), Singapore 188976, Singapore; 9 Language and Communication Centre, School of Humanities and Social Sciences, Nanyang Technological University, Singapore 639798, Singapore; 10 Policy Wisdom LLC, Quebradillas 00678-2705, Puerto Rico; 11 Health Technology Assessment Unit, Cancer Research Department, Arturo López Perez Foundation, Santiago 7500921, Chile; 12 School of Medicine, Universitat Autònoma de Barcelona, 080193 Barcelona, Spain; 13 Faculty of Pharmaceutical and Chemical Sciences, University of Chile, Santiago 8380000, Chile; 14 European Reference Network for Hereditary Metabolic Diseases (MetabERN), 33100 Udine, Italy; 15 Regional Coordinating Center for Rare Diseases Friuli Venezia Giulia, Udine University Hospital, 33100 Udine, Italy; 16 Brains for Brain Foundation, 35128 Padova, Italy; 17 The Translational Science Training Program, National Institutes of Health (NIH), Maryland, MD 20814, USA; 18 Children’s National Medical Centre, Washington, DC 20010, USA; 19 National Organization for Rare Disorders (NORD), Quincy, MA 02169, USA; 20 Children’s National Rare Disease Institute, Washington, DC 20012, USA; 21 Department of Pediatrics, George Washington University, Washington, DC 20052, USA; 22 Thai Rare Disease Foundation (ThaiRDF), Bangkok 10230, Thailand; 23 Prader-Willi Syndrome Association (PWSA) of Thailand, Department of Pediatrics, Faculty of Medicine, Ramathibodi Hospital, Mahidol University, Bangkok 10400, Thailand; 24 Rare Disease Working Committee, Thai National Health Security Office (NHSO), Bangkok 10210, Thailand; 25 Sub-Working Committee for Rare Disease Medicine, Thailand National List of Essential Medicines (NLEM), National Drug Policy Division, Food and Drug Administration, Nonthaburi 11000, Thailand; 26 Medical Genetics Network, Genetics Society of Thailand, Bangkok 10330, Thailand; 27 Thailand Medical Genetics and Genomics Association (TMGGA), Bangkok 10510, Thailand; 28 Asia Pacific Society of Human Genetics (APSHG), Singapore 229899, Singapore; 29 Department of Pediatrics, Faculty of Medicine, Ramathibodi Hospital, Mahidol University, Bangkok 10400, Thailand

**Keywords:** rare diseases, burden of disease, patient journey, social impact, disease management, health policies, health equity

## Abstract

This document provides a comprehensive summary of evidence on the current situation of rare diseases (RDs) globally and regionally, including conditions, practices, policies, and regulations, as well as the challenges and barriers faced by RD patients, their families, and caregivers. The document builds on a review of academic literature and policies and a process of validation and feedback by a group of seven experts from across the globe. Panelists were selected based on their academic merit, expertise, and knowledge regarding the RD environment. The document is divided into five main sections: (1) methodology and objective; (2) background and context; (3) overview of the current situation and key challenges related to RDs covering six dimensions: burden of disease, patient journey, social impact, disease management, RD-related policies, and research and development; (4) recommendations; and (5) conclusions. The recommendations are derived from the discussion undertaken by the experts on the findings of this review and provide a set of actionable solutions to the challenges and barriers to improving access to RD diagnosis and treatment around the world. The recommendations can support critical decision-making, guiding efforts by a broad range of RDs stakeholders, including governments, international organizations, manufacturers, researchers, and patient advocacy groups.

## 1. Introduction and Context

The definition of rare disease (RD) varies across the world. According to a systematic global review, there are about 296 definitions from 1109 organizations, most of which are explicitly or implicitly derived from prevalence thresholds. Such definitions are expressed as a fraction, a percentage, or the number of cases per population (whether that be from 10,000 or 100,000 people) [1]. Only a few countries consider a broader set of dimensions that are relevant from a healthcare policy perspective, capturing aspects of vulnerability and socioeconomic impact. This is, for example, the case in Thailand, where the RD definition used by the Universal Coverage Scheme is derived from a combination of a number of cases per population and a broader set of qualitative descriptors that include severity (defined by high morbidity and premature death); difficulties accessing adequate diagnosis, screening, treatment, and lifelong treatment; and high economic burdens on the family and society [2]. 

In response to the need to have a common and comprehensive definition of RD, in December 2019, Rare Diseases International (RDI) signed a landmark agreement with the World Health Organization (WHO) to, among other priorities, develop an operational definition of RD that can be used globally [3]. In contrast to definitions that are often shaped by regulatory processes and prioritized disease prevalence thresholds and frequency, the operational definition of RD proposed by this initiative considers the specific clinical and qualitative challenges associated with the low prevalence of the conditions, providing a framework that reflects what diseases are considered rare, how many people are affected, and why the RD community requires specific attention [4].

“*People living with RDs face distinct and significant challenges that arise from the infrequency of their medical conditions, such as a long diagnostic journey, inadequate clinical management, and limited access to effective treatments. The burden of RD on patients, their carers and families, healthcare systems, and society overall, merits greater visibility and recognition. An RD is a medical condition with a specific pattern of clinical signs, symptoms, and findings that affects fewer than or equal to 1 in 2000 persons living in any WHO-defined region of the world. RDs include, but are not limited to, rare genetic diseases. They can also be rare cancers, rare infectious diseases, rare poisonings, rare immune-related diseases, rare idiopathic diseases, and rare undetermined conditions. While the frequency of most rare diseases can be described by prevalence, some RDs, such as rare cancers and rare infectious diseases, can be more precisely described by incidence.*”

[4]

A common operational definition is a strategic step to respond to the need of the RD community to join forces, as well as a recognition of a common struggle. There are about 5000 to 8000 RDs identified globally [5], varying regarding their origins, risk factors, symptoms and treatments, and geographical dispersion [6]. Despite this wide variety of specificities, they share the commonality of being rare, with patients facing similar challenges regarding diagnosis, treatment, and care.

People living with RDs often struggle with social and cultural exclusion, limited political visibility and prioritization, and difficulties accessing adequate diagnosis, treatment, and care. The lack of epidemiological data that is consistent across geographies and populations and the nature of the rarity of the conditions (low prevalence) also limit the implementation of clinical trials and the gathering of real-world data. This, in turn, hinders the production of effective and innovative treatments. The management of diagnosis and treatment of RDs is also challenging. Patients, their families, and their caregivers are often affected by the heavy emotional, financial, and social burdens, and although strong disparities between countries exist, RD management remains a global concern. Even in developed economies that have more advanced RD policies, budgets, and clinical guidelines, patients struggle to navigate the healthcare system, leading to major disparities in RD quality of care and access to treatment across social classes and communities [7]. 

## 2. Methodology and Objective

This document seeks to provide a comprehensive summary of evidence on the current situation of RDs globally and regionally, including current conditions, practices, policies, and regulations, as well as the challenges and barriers faced by RD patients, their families, and their caregivers. Based on the understanding of the current situation, the document identifies the key areas where action is needed to resolve the main challenges and barriers and provides a set of actionable solutions (recommendations) to improve access to RD diagnosis and treatment services around the world. By illustrating a path forward, the recommendations can support critical decision-making, raise awareness, and guide advocacy initiatives involving a broad range of stakeholders, including governments, international organizations, manufacturers, researchers, and patient advocacy groups (PAGs). 

The methodology employed to build this document includes a review of literature and policies and a process of validation and feedback with a group of seven experts from across the globe. Panelists were selected based on their academic merit, expertise, and knowledge regarding the RD environment. Their disciplinary backgrounds include pediatrics, genetics, economics, and public health, among others. Representation of expertise across the different geographic zones was considered essential.

Global, regional, and country-level evidence were collated and analyzed between 1 April 2021 and 4 August 2022. To showcase different contexts and scenarios, an analysis of regional evidence was performed according to five zones: North America, Latin America, Europe, Asia-Pacific, and Africa, and the Middle East. Data and evidence on RDs came from a myriad of resources. Peer-reviewed pieces were prioritized whenever possible. 

Sources were selected and prioritized to capture the current situation and challenges related to RDs across six topics or dimensions:

(1) Burden of disease, including epidemiological and economic impact;
(2) Patient journey, including availability and access to diagnosis and treatment and impact on quality of life;
(3) Societal impact, including impact on patients, their families, and their caregivers, comprising professional, educational, and social life, and psychological and emotional wellbeing;
(4) Disease management, including the strategies and programs available to manage patients along the continuum of care;
(5) RD-related policies, including global, regional, and national plans, strategies, and measures that impact (whether positively or negatively) RD diagnosis and treatment;
(6) Research and development, including challenges in developing evidence across different study designs.

Literature was collected through a targeted search covering the six topics or dimensions for each of the geographic zones. It is important to note that studies and literature of national scope were also considered. This was particularly important to capture and showcase how conditions and challenges are present in different contexts. Relevant literature was also identified and shared by the experts during the offline review process. 

The findings were gathered, synthesized, and appraised to ensure quality. The information gathered was used to produce a working document. The working document organized findings according to the dimensions and geographic zones, providing first a narrative on the current situation, followed by a synthesis of the challenges, except for research and development (as challenges were deemed common across regions). The experts discussed and validated the evidence during an online panel session hosted on 21 September 2022, followed by multiple rounds of offline review and calls until finalized. 

## 3. Current Situation and Key Challenges Related to RDs

In this section, we will provide a comprehensive summary of findings regarding the current situation and the challenges across six pre-established dimensions. Each section (besides research and development) is organized in a similar manner, first introducing an overview of the current situation globally and from a regional perspective, then reflecting and summarizing the main challenges identified through the review of literature and evidence. The section on research and development has a different organization as the issues and challenges regarding how to develop evidence on RDs are transversal across regions as they associate with the methodology and study design. This section is instead organized by type of study design, providing an overview of the limitations and challenges in each case.

### 3.1. Burden of Disease

The burden of disease refers to the impact of health problems on a given population, typically considering clinical, economic, and/or political indicators and often expressed in terms of the costs of disease to individuals, healthcare systems, and/or society [8]. Estimating the global burden of RDs is a challenging task for at least three reasons: the coexistence of multiple definitions across the globe, the diversity of conditions that the concept encompasses, and the limited capacity to systemically track and diagnose these populations in several countries and regions. Nonetheless, understanding the burden of RDs is fundamental to informing public policy and defining priorities [6]. 

#### 3.1.1. The Epidemiological and Economic Impact at the Global Level

According to a report from the World Economic Forum published in 2020, it is estimated that between 350 and 475 million people are affected by RDs globally [9], many of whom are children (approximately 50% of the people affected by RDs) [10]. This has significant consequences both in terms of lives lost and the social and economic burden on families and caregivers. It is estimated that RDs are responsible for 35% of deaths in the first year of life [11] and that 30% of children with an RD will not live to see their fifth birthday [10]. Caring for RD patients is time-intensive, emotionally stressful, physically demanding, and economically straining [12,13,14]. Whether due to the impact on patients’ and caregivers’ capacity to work or the high out-of-pocket expense of RD treatments, many families struggle to make ends meet [15,16,17,18]. 

The economic impact of RDs is large and includes direct costs of treatment, along with non-clinical supporting costs and an overall cost of lost productivity for the patient and their caregivers. According to a meta-analysis of studies published between 2010 and 2017 (mostly from North American or European countries), direct health costs account for most of the economic impact of RDs, with great cost variability across RDs and countries [19]. A systematic review of cost-of-illness studies for RDs found scarce evidence and high variability across conditions. For example, the total cost per patient per year for haemophilia was estimated to range from EUR 1101 to EUR 178,796, making it difficult to compare to the economic impact of other common conditions [20].

The high economic impact of RDs is closely linked to the costs associated with drugs and care. Some of the most expensive drugs on the market are targeted to treat RDs [21]. Orphan drugs cost roughly five times more than non-orphan drugs [22], which poses challenges and concerns in terms of the sustainability of health systems [23]. However, the high economic impact of RDs is also driven by the cost of care. Evidence indicates that costs are higher in a scenario without treatment when compared to a scenario with treatment [24]. 

Considering the estimated prevalence of 100,000 people, the most common RDs globally are narcolepsy (50), primary biliary cholangitis (40), Fabry disease (30), cystic fibrosis (25), hemophilia (20), spinal muscular atrophy (13), and retinal dystrophy (13) [25]. 

#### 3.1.2. The Epidemiological and Economic Impact per Region

**Figure 1:**
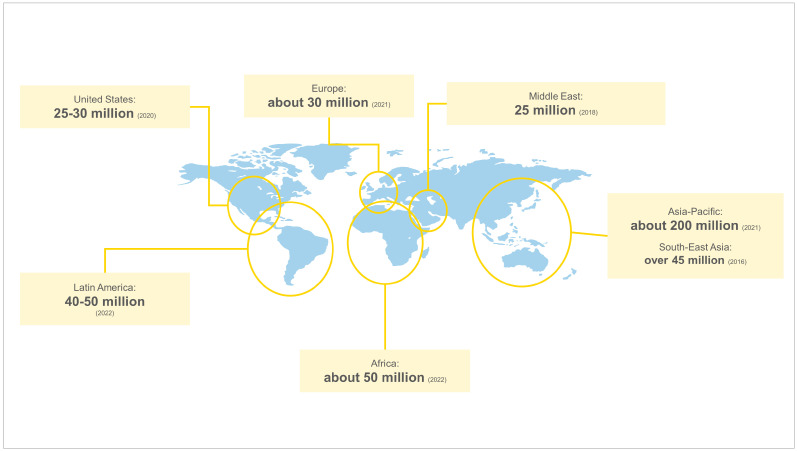
Number of people affected by RDs across regions. Source: Elaborated by authors based on overviewed literature [26,27,28,29,30,31,32,33,34]. The estimated values depend on data availability and local diagnostic and tracking capacity.

Many countries have only estimations for prevalence data available, as RDs are commonly under-diagnosed. Figure 1 presents the estimated number of people affected by RDs across regions. Asia-Pacific is the region with the highest estimated absolute value of people affected by RDs, with around 200 million people living with these conditions [26]. Anecdotal evidence suggests that this might be linked to the population size of the region (which is the largest), as well as practices present in some countries (such as consanguineal marriages and maternal and paternal advanced age). Africa has around 50 million people affected by RDs [27], similar to Latin America, where an estimated 40 to 50 million people are living with an RD [28,29]. Among countries in the region, the highest burdens are observed in Brazil, Mexico, and Argentina [30]. RDs affect around 30 million people in Europe [31], between 25 and 30 million people in the United States [32,33], and 25 million people in the Middle East [34].

The prevalence of RDs varies across geographic areas due to population genetic diversity, as well as environmental and behavioral factors [35,36,37]. However, it is important to note that the true prevalence of RDs depends on standardized, well-established, and specific diagnostic criteria, which vary across countries and regions [38]. Low-resource settings may face limited clinical information, lack of reliable epidemiological data, inappropriate diagnostic knowledge and resources, and poor monitoring systems [39], leading to an underestimation of RD prevalence. Compared to Europe or the United States, evidence indicates a lack of RD epidemiological information in many parts of the world, such as India, China, South America, and Africa [6]. Acknowledging this reality is particularly important when comparing RD prevalence among and within regions. In the African continent, for example, it may be reasonable to speculate that a higher prevalence of RDs in South Africa, when compared to neighboring countries, could be associated with having a more developed tracking system, although we found no robust evidence to support this claim.

In North America, data from the United States highlights the high economic burden of RDs and how it associates to direct as well as indirect and non-medical costs. RD expenditure in the USA was estimated to be USD 966 billion in 2019, from which USD 418 billion corresponded to direct medical costs and USD 548 billion to indirect and non-medical costs [40]. To put this into perspective, these values are higher than those estimated for some chronic diseases, such as diabetes (USD 966 billion for 15 million RD patients vs. USD 327 billion for 24.7 million diabetes patients) [40]. The leading categories for direct medical costs include inpatient and outpatient services, other ancillary services, and prescription drugs. For indirect and non-medical costs, the leading categories are absenteeism, presenteeism, forced retirement, and healthcare services not covered by insurance. Nonetheless, while direct healthcare costs are high, the cost of treating RD patients might be even higher. A systematic review of the costs of sickle cell disease (SCD) and treatments in the United States found that long-term treatment of SCD can decrease total medical costs, as complications lead to increased hospital visits and healthcare utilization [41].

Furthermore, the financial burden incurred due to out-of-pocket RD treatment spending might put patients and their families in economic vulnerability. Considering that the median household income in the United States was USD 67,521 in 2020 [42] and that out-of-pocket RD treatment costs were estimated at approximately USD 26,887 per-person excess cost when compared to a person without an RD [40], the medical costs an average RD patient faces represent approximately 39% of the total median household income.

In Latin America, evidence also demonstrates the economic burden of RDs due to direct and indirect costs on health systems. For example, in Mexico, the direct and indirect annual cost per patient with hemophilia, including diagnosis, follow-up, prophylaxis, treatment, and hospitalization, was estimated at USD 332,458 in 2019, out of which a substantial share depended on the use of hemostatic factors (the annual cost per patient with inhibitors was 4.2 times higher than that for patients without inhibitors) [43]. Like North America, the high direct economic burden is often driven by the high costs of medicines. For example, in Ecuador, it was estimated that the treatment per patient with hemophilia had a monthly cost of approximately USD 13,172 in 2017 [44]. Aside from direct costs, patients and their families often shoulder significant indirect and intangible costs associated with RDs. This is, for example, the case of patients with mucopolysaccharidosis VI in Colombia, who face the responsibility of costs associated with complications, frequent healthcare encounters, and caregiver dedication [16]. Patients with RDs, although not very numerous, also represent a high economic burden on countries’ national budgets. For example, in Peru, the Ministry of Health spent USD 33 million in care for over 42,000 people living with a rare or orphan disease in 2019 [45], and in Colombia, the economic burden of atypical hemolytic uremic syndrome treatment was USD 3,907,891 for only 18 patients in 2019 [46]. 

In Europe, one study published in 2016 analyzed the social and economic costs of RDs and estimated that drugs represented nearly 90% of direct healthcare costs (costs attributable to patient care, medical management of the disease, drugs, admissions, and complementary tests) in most of the analyzed countries [47]. In fact, the share that orphan drugs represent in relation to total pharmaceutical expenditure has increased since 2000 across European countries. Orphan drugs expenditure reached approximately EUR 10.5 billion in 2017, while the total value spent on medicines that year was approximately EUR 147 billion, which represented 7.2% of total pharmaceutical expenditure (this share was around 4% in 2012 and less than 1% in 2005) [48]. While orphan drug designation and marketing authorization are centralized in the European Union (EU), decisions on pricing, reimbursement, and funding for orphan medicines remain the responsibilities of Member States. This leads to uneven access to orphan medicines across Europe and a great variation in the costs of orphan drugs per patient [49]. The differences in costs per RD patient across different European countries demonstrate that market access strategies are fragmented, impacting orphan and non-orphan medicines [50]. This, in turn, affects the quality of care and treatment that RD patients receive [51,52].

In Asia-Pacific countries, the affordability of drugs to treat RDs is one of the major issues faced, as the strain put on households by out-of-pocket RD treatment costs places patients and their families in a particularly vulnerable position, with many families being unable to afford treatment. In Australia, a study found that 45% of parents of children who have an RD are not able to cope with the costs associated with their children’s conditions, and 29% had to increase their working hours or take a second job [17]. In China, one study found that over 90% of RD patients could not afford their costs of living in 2016 and that the medical expenditure of an individual with an RD was, on average, three times higher than his or her individual income [18]. Moreover, in some countries, the lack of local production of RDs medicines and heavy reliance on imported drugs lead to higher costs of treatment, posing a higher financial burden on RD patients and health systems. This is, for example, the case in India [53,54]. Finally, like other regions, evidence from some countries also indicates that drug expenditures account for most of the direct RD healthcare costs. For instance, in Taiwan, a 2019 study not only revealed that drug expenditures for the treatment of RDs increased from USD 13.24 million in 2003 to USD 121.98 million in 2014 (accounting for 2.31% of drug expenditures for the total population), but also that expenditure accounted for 70% and 89% of the total health expenditures for patients with RDs, respectively [55]. 

In Africa and the Middle East, evidence is indicative of the need to have policies for RDs that are comprehensive and multidimensional. In this region, RDs are low on the health policy agenda, as demonstrated by the presence of underdiagnosis, lack of care, and lack of evidence, which result in substantially higher costs down the line [56]. Moreover, in many countries, competing priorities, such as nutrition and communicable disease prevention, constrain the possibilities of health systems to provide adequate care for RD patients. For example, in South Africa, even though it is estimated that one in 15 people are affected by an RD [57], directing the additional funds necessary to adequately manage RDs is limited by the lack of public investment in health [57]. Like other regions, in the Middle East, patients with RDs face high costs to access medicines. For instance, in Saudi Arabia, the reimbursement system severely limits the coverage for orphan drugs, which, together with bureaucracy on imported goods, delays treatment and leads to disease deterioration [58]. 

#### 3.1.3. Gaps, Barriers, and Challenges

RDs are tremendously heterogeneous in their symptoms, progression, how they affect patients, and potential treatments. Due to the disease heterogeneity and geographic dispersion, there is a lack of reliable and significant evidence on their impact globally. Moreover, evidence of the economic impact of these diseases is still missing in many parts of the world. Most countries in the Asia-Pacific, Africa and the Middle East, and Latin America lack or have limited evidence of the economic impact of RDs.

The high costs of medicines represent a substantial share of the economic burden of RDs. There are methods and tools that can be employed to control costs, such as repurposing drugs for new indications. This is a timesaving, cost-efficient method that can accelerate the development of RDs treatments [59]. A successful case of drug repurposing is the use of Gentamicin (an aminoglycoside antibiotic used in the treatment of several gram-negative infections) to treat Duchenne muscular dystrophy [60]. Clinical studies have demonstrated its effectiveness and long-term safety in treating this RD [61]. Despite its potential, this approach can be hampered by different challenges such as financial and intellectual property considerations, the regulatory path, and challenges in performing clinical trials [60].

In a general sense, there is a paucity of cost-of-illness studies on RDs [62]. The limited availability of medical history and epidemiologic data regarding RDs, as well as a standardized methodological approach to calculate cost-of-illness, constrains the possibility to estimate direct and indirect costs associated with RDs and, consequently, the estimation of potential economic benefits of treatment [63,64]. Moreover, cost-of-illness studies of RDs rarely analyze the outcomes or benefits of possible treatments [65]. While this is true, evidence also indicates that economic benefits derived from RD therapies will likely manifest in the long term, such as reducing both direct and indirect expenditures [64]. One study on direct, indirect, and mortality-related costs for a sample of 24 RDs in the United States found that total costs per RD patient per year were 21.2% higher in a scenario without treatment, when compared to a scenario with treatment, highlighting the substantial value that access to RD treatment generates [24]. 

There is also a lack of reliable, consistent, and multidimensional socioeconomic measures on the burden of disease to capture the full scope of the impact of RDs on patients, their families, their caregivers, and society at large. The absence of a common measure further limits the possibility of assessing the value of RD innovation using a multi-criteria approach (beyond cost-effectiveness). Decision-making regarding the assessment, financing, and reimbursement of RD innovation requires robust evidence-based analyses [8]. Likewise, policy and budget planning to treat, diagnose, and care for RD patients also requires real-world data. The lack of interoperable RD surveillance systems and registries in many regions of the world (including the Asia-Pacific, Africa and the Middle East, and Latin America regions) particularly limits the capacity of decision makers to respond to the needs of the RD community. Overall, it is crucial to collect and communicate evidence on the burden of RDs that is compelling to regulators, policymakers, and payers, so they are equipped to understand the scale of the issue, acknowledge the needs of the population, and make decisions accordingly [66]. 

Regarding funding, many countries continue to struggle with putting RDs on the policy agenda. This is especially true for lower-income countries with a high prevalence of communicable diseases. As for high-income countries, the main issue rests on the lack of organization of different funding programs, as well as a lack of dedicated RDs research funds [67]. With few incentives and little support available for RDs research, decision makers struggle to measure and understand the return on investing in RDs. 

Finally, the increasing number of novel RDs being identified requires a more holistic focus by patient organizations, to ensure patients with novel RDs count with the community support needed. According to evidence, approximately 50% of RDs do not have a disease-specific foundation/research group or community readily available [68]. Patient organizations play a pivotal role in advocating for the development of new therapies. There is a need to continue empowering patient organizations to participate knowledgeably during reimbursement decision-making. 

### 3.2. The Patient Journey

**Figure 2:**
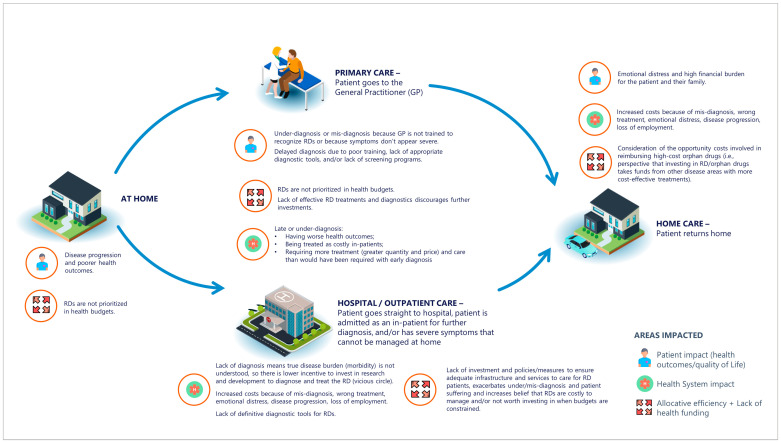
Challenges along the RD patient journey. Source: Elaborated by authors.

The patient journey represents the sequence of events that a patient experiences within a healthcare context from the onset of disease to diagnosis and having their needs addressed and their disease managed. Despite RDs typically exhibiting substantial clinical heterogeneity, RD patients share similarities regarding the challenges they and their families face at various points of care. Figure 2 summarizes some of the main steps during the patient journey, highlighting the challenges faced along the way. While the graph clearly represents the challenges, it is important to acknowledge the pivotal role that health policies, investment, and government commitment play in ensuring that primary care and hospitals provide adequate services regarding diagnosis, treatment, and care for RD patients.

#### 3.2.1. Global Overview of the Patient Journey

According to our findings, the challenges faced by RD patients during the care journey can be organized into four groups: challenges regarding diagnosis, treatment, overall life quality, and financial protection (see Table 1). In a general sense, arriving at an accurate diagnosis is the first gatekeeper to access treatment and care. Evidence worldwide indicates that diagnosis is often a lengthy journey, with many patients having to endure multiple interactions with specialists and healthcare providers to be accurately diagnosed [69,70,71,72]. Even when treatment is available, delayed and/or wrong diagnosis prevents patients from accessing the treatment and care they need [66,69,70,71,72,73,74,75]. 

However, access to accurate and timely diagnosis is only one of the many hurdles to treatment. The first and perhaps most obvious is the lack of approved medical treatments for some RDs [66,74,76]. Evidently, more research and investment are needed to develop treatments for newly identified RDs. Evidence from across the world also confirms that, even when existing, RD patients often struggle to access treatment due to disparities between rural and urban settings (as evidenced by other, more common diseases). Many RD patients are forced to travel long distances (sometimes even outside their own countries) to access treatment and shoulder the associated costs [58,67,69,77,78,79]. Having to navigate different doctors, specialties, and healthcare providers is not an easy task, especially in contexts that lack interoperational healthcare systems [69,70].

RD patients and their families are therefore at higher risk of experiencing poor quality of life, increased mental health issues, more social isolation, and poor work-life balance [14,69,76,80]. Notably, the impact RDs have on the lives of patients and their caregivers is further exacerbated by the lack or limited reimbursement of orphan drugs and the consequent burden of out-of-pocket expenses on RDs households [69,81,82,83]. While the next section will include a more detailed account of the social and psychological impact of RDs on patients and their families, it is important to highlight the need for a more comprehensive body of RD-related policies that can respond to the multidimensional needs of support and care.

**Table 1:** Overview of challenges faced by RD patients along the patient journey.

	Diagnosis	Access to Treatment	Quality of Life	Financial Protection
Key take aways	Delayed diagnosis. Wrong diagnosis. Multiple interactions with specialist/HS before diagnosis.	Disparities in available/accessible medical treatments between rural and urban settings. Need to travel to access treatment. Difficulties navigating fragmented healthcare system. Lack of approved medical treatment.	High risk of experiencing poor QoL. High risk of experiencing mental health issues and sense of isolation (patients and caregivers). Negative effect on work-life balance.	Lack of reimbursement of orphan drugs. Considerable out-of-pocket expenses.
North America	Delayed diagnosis [73]. Multiple interactions with specialist/HS before diagnosis [40].	Difficulty accessing treatments/therapies for symptom management [76]. Difficulty accessing treatment for the underlying disease [12,76]. Age-related inequalities accessing RD treatment [12].	High risk of experiencing poor QoL [76]. High risk of experiencing mental health issues [76].	Lower-income households experience higher financial burden and access barriers to treatment [81].
Latin America	Delayed diagnosis [70]. Wrong diagnosis [69,71]. Multiple interactions with specialist/HS before diagnosis [69,71].	Transportation and long journeys to access treatment [69]. Difficulties navigating fragmented healthcare system [69,70].	Anxiety associated with not having enough information, a definitive diagnosis, and issues accessing treatment [69].	Available drugs are often not reimbursed [69].
Europe	Delayed diagnosis [72]. Wrong diagnosis [72]. Multiple interactions with specialist/HS before diagnosis [72].	Disparities in available medical treatments across countries [77]. Need to travel to access treatment [78]. Limited production capabilities and supply of raw materials and long manufacturing process (according to manufacturers) [84].	Risk of experiencing mental health issues (patients and caregivers) [14]. Negative effect on work-life balance [14].	Reimbursement of and access to orphan drugs varies across countries [85].
Asia-Pacific	Delayed diagnosis [67,74].	Lack of approved medical treatment [67,74]. Long waiting times to access government-funded treatment [86]. Need to travel to access affordable treatment [67,79].	Risk of experiencing mental health issues (patients and caregivers) [80]. Risk of experiencing sense of isolation [80].	Lack of reimbursement of orphan drugs [80]. Considerable out-of-pocket expenses [80].
Africa and the Middle East	Delayed diagnosis [75]. Wrong diagnosis [75].	Disparities in available medical treatments between rural and urban settings [58]. Delayed importation of medicines due to bureaucracy [58].	Lack of prioritization, facilities, support, and care beyond the family circle [7,56]. Significant increase in orphans might overwhelm kinship networks, government programs, and the community [56].	Lack of reimbursement of orphan drugs [7,83]. Lack of availability of orphan drugs [7].

Elaborated by authors based on overviewed literature [7,12,14,56,58,69,70,71,72,73,74,75,76,77,78,79,80,81,82,83,84,85,86].

According to RDI, there are ten areas governments and decision makers across the globe can consider to positively impact access to quality and timely diagnosis, treatment, and care for RDs [7]:

- Social and cultural acceptance, equality, and inclusion of persons living with an RD.
- Systematic, standardized data collection and data sharing.
- Political recognition and a dedicated policy framework and budget for RDs.
- Availability, affordability, and coverage of RDs tests and medicines.
- Focus on prevention and screening.
- Widespread availability of expertise, specialized services, and standards of care.
- Coordination of care across devolved or fragmented healthcare systems.
- Geographical and cultural considerations.
- Support for technology infrastructure and use of telemedicine.
- Empowerment of patients and families to self-care and advocate.

The ten areas aim to serve as a guide that governments and decision makers can use according to their needs. How they are prioritized will likely vary according to the robustness of healthcare systems, the specific needs of the condition, and the maturity of the advocacy community [7].

#### 3.2.2. Regional Overview of the Patient Journey

Even in well-organized countries with comprehensive health systems, such as the ones in North America, RD patients face long waiting periods before being diagnosed, often needing several interactions with the healthcare system. A survey conducted by the Canadian Organization for Rare Disorders found that about 20% of patients wait between 6 and 14 years to get diagnosed, and 60% of them consult 3 to over 20 specialists before receiving a diagnosis [73]. Similarly, in the United States, RD patients need an average of 17 interactions with the healthcare system to arrive at a proper diagnosis [40]. To have access to treatment is also not necessarily straightforward. A study in the United States found that roughly one out of every three caregivers report having difficulties in accessing treatments or therapies for symptom management or treatment. The main reason identified by the caregivers was the lack of available treatment (only 43% reported having a medicine available for the RD) [76]. Access to treatment also seems to disproportionally affect the younger and the poorer segments of populations, similar to what happens regarding the treatment of other diseases [87,88]. One study from the United States found that fewer children access RD treatment and take prescription medication when compared to the adult population [12] and another found that insurance policies and off-label prescribing were especially discriminatory for lower-income patients. It was discovered that individuals earning less than USD 20,000 per year were twice as likely to be denied referral to a specialist as those earning USD 100,000 or more [81]. This situation, however, is not exclusive to RD patients, as there is evidence of significant differences in access and affordability by income in the United States overall [89].

Similarly, in Latin American countries, RD patients often face a long and tortuous journey before receiving a diagnosis and treatment plan, with challenges persisting when accessing ongoing care. RD patients and caregivers in Brazil, Colombia, and Argentina typically report a history of many interactions with health facilities and different specialties, wrong or late diagnosis, and lack of or inadequate treatment [69]. In Colombia, patients have at least eight consultations before a final diagnosis and receive at least three wrong diagnoses [71]. In Argentina, RD patients and their families reported that the most common causes of diagnosis delay are related to physicians (e.g., lack of medical knowledge, lack of medical commitment, and isolated consideration of symptoms) [70]. Moreover, even with an adequate diagnosis, RD patients face access issues, as they must travel to different hospitals for specific tests, with transportation and long journeys being some of the main barriers faced [69]. In Argentina, around 50% of patients consult three or more centers, many of which require traveling to other towns or provinces [70] since the large urban centers contain most of the healthcare facilities in the country [90].

Because patients and caregivers often lack a general understanding of genetic diseases, their quality of life is negatively impacted by the anxiety associated with not having a definitive diagnosis and treatment [69]. In many countries of the region, patients and their families are forced to undergo agonizing legal battles to access treatment. In Brazil, for example, where orphan drugs are often not reimbursed, RD patients usually engage in legal proceedings (judicialization) to obtain access to/reimbursement of certain drugs. This bureaucratic burden is exacerbated by the medical reports needed during the proceedings [69].

In Europe, there are great inequities across regions and countries in the availability, access, and reimbursement of RD treatments. Regarding diagnosis, studies report that one in four RD patients face between 5 to 30 years to obtain a diagnosis [72,91], and one in four RD patients must travel to a different region to be diagnosed [72]. In more than 40% of cases, patients receive a misdiagnosis more than once [92]. Despite advances in the knowledge of some diseases and innovations in terms of health technologies, late diagnosis is a persistent challenge for RD patients. In the Netherlands, for example, the time to diagnosis for patients with mucopolysaccharidosis I and III has not changed since 1988 [93]. 

RD patients in Europe also face access barriers regarding the availability of treatment, with nearly a quarter of them not receiving treatment because it is not available in their country [77]. Moreover, even when available, it is usually not geographically accessible, with many being forced to travel outside their region. This is particularly problematic as many RD patients have difficulties with mobility [78]. A 2017 survey by the European Organization for Rare Diseases (EURORDIS) confirmed that 24% of RD patients did not receive treatment due to the lack of availability in their country (vs. 7% of the general population) and 15% due to difficulties affording treatment (vs. 6%) [85]. A study of the economic burden of RDs in the United States discussed that when taken together, indirect (labor market productivity losses due to RDs, including absenteeism, presenteeism, and earnings losses from forced retirement) and non-medical costs exceed direct medical costs [40]. It is, however, difficult to assess if this finding can be extrapolated, as studies are often not large enough in terms of scope and do not truly reflect the complexity of all RDs and their impact [8]. 

While the EU has taken important steps to centralize regulatory processes, leading to RDs drugs being approved simultaneously for all Member States, health technology assessment (HTA), pricing, and reimbursement remain challenges. Decision-making in this respect remains the responsibility of countries, which means differences in patient access persist across the region [85].

In the Asia-Pacific region, the main challenges regarding the RD patient journey include lack of training of health professionals, facilities, and reimbursement of treatments. There is a lack of clinical RD expertise across the region, and only a few health centers offer specific services to treat RDs. For example, in Thailand, a study from 2016 reported that there were only 22 geneticists available (most of which were located in major cities) to serve a population of 67 million people [80]. Patients with RDs across the region also encounter barriers to accessing treatment and care given the limited funding by the government [80]. This is the case even in some developed economies. For example, in Australia, patients wait between two and four years longer to access government-funded treatment for RDs when compared to similar countries [86]. Furthermore, there are cases where medicines that can be used to treat an RD are only subsidized under the Pharmaceutical Benefits Scheme for a common disease, meaning RD patients must pay more for the same medicine [86]. 

Despite progress regarding the development of new drugs, effective and safe treatment is not available for most RDs [74,80]. However, like in other regions, even when available, approval processes might delay access. For example, a study found that 43% of the orphan drugs approved in the United States were not approved in Japan [94]. This means that patients are often forced to travel both internally and abroad to seek the help they require [67,79].

The feeling of being affected by an unusual, poorly understood disorder can bring a sense of isolation to RD patients and their families [67,80]. The lack of reimbursement of orphan drugs by the public health systems creates greater inequities across populations. A study conducted in South Asia found that most people with low incomes are unable to access treatment [80]. A survey conducted in 2020 to assess RD awareness and management in Asia-Pacific, including a diversity of stakeholders, found that 47% of respondents identified improving financial support as the single most impactful action to enhance the lives of those living with RDs [67].

In Africa and the Middle East, the lack of capacity and resources to diagnose, bureaucratic delays, and disparities between the rural and urban settings are the main factors that hinder the quality of care and treatment of RD patients. Both the public and private sectors lack the capacity and resources to diagnose RDs [82]. The delay in diagnosis leads to the progression of the disease and worse health outcomes. In Egypt, for example, myeloproliferative neoplasms are often diagnosed at an advanced stage due to difficulties differentiating this disease from more common conditions [75]. Access to treatment is hindered both by the geographic context (RD care and treatment are concentrated in urban areas) [58] and the lack of availability of orphan drugs and gene therapy. For example, in Saudi Arabia, bureaucratic proceedings regarding imported pharmaceuticals delay treatment plans [58]. The poor quality of life of RD patients in countries in Africa and the Middle East is exacerbated by the lack of prioritization, facilities, support, and care beyond the family circle [56,82], many of which are common to those suffering from other conditions or requiring care. For example, evidence indicates that families in sub-Saharan Africa are undoubtedly the main providers of elder care [95]. Patients with RDs in these countries are also affected by the unavailability and lack of reimbursement systems for most orphan drugs [82,96]. 

#### 3.2.3. Gaps, Barriers, and Challenges

When discussing the patient journey for people living with RDs, the numerous challenges regarding diagnosis, care, and treatment render their experiences more comparable to an odyssey. While some countries such as Australia, France, Italy, Japan, and the United States have established dedicated RD diagnosis programs to speed up the process of diagnosing patients with RDs [97,98,99,100,101], this is not the case for most countries. 

RD patients frequently face a critical lack of information and support, often resorting to local patient organizations for help. Patient organizations could support patients facing such odyssey by providing institutional information tools (such as websites and helplines), as well as social and psychological services [102]. Patient organizations also play a critical role in advocating for a more favorable policy environment for RDs. The active involvement of patients and patient organizations in regulatory and commissioning processes is valuable, holding the potential to expedite patient therapeutic access [103]. Patient organization availability, capabilities, and capacity to fulfill these roles are critical.

The image depicted by the regional overview highlights the great disparity across and within countries in accessing basic RD diagnosis, treatments, and care. Treatment is not available everywhere, with considerable gaps between rural and urban settings, and it is not uncommon for RD patients and their caregivers to be forced to permanently relocate to access care. Moreover, patients with RDs often struggle to find physicians knowledgeable about their conditions, leading to delays in the correct diagnosis and, subsequently, adequate treatment and care. Diagnosis and ongoing care are often provided by multiple health centers, which brings challenges regarding access and complicates RD patients’ journeys. 

New therapies/interventions are costly, and orphan drugs are not reimbursed in all countries, putting RD patients in a particularly vulnerable position. There are many tools available for resource-limited settings to reduce the costs of drugs, including partnerships and programs in collaboration with key stakeholders (such as the industry) and approaches (often used for procurement purposes) to reduce costs. One example is of a drug made available to patients with chronic myeloid leukemia in some African countries through pharmaceutical and foundation partnerships [104]. While this approach can be successful at reducing costs, it is conditional to the priorities set by the donors [105]. Decision makers can also use procurement mechanisms to reduce the costs of drugs, such as price control, centralized purchasing, and prioritizing value (value-based procurement). Although these tools might prove to be successful at reducing costs, it is important to note that they are not exclusive to RDs, nor are they common in lower-resource countries (where they might be needed most) [106]. 

The development of orphan drug policy on a global scale remains limited due to challenges in generating accurate evidence to sustain and justify the change. Efforts to prioritize RDs on public health and policy agendas are often overlooked by governments, and most countries lack the systems, mechanisms, and coordination to develop people-centered policies, treatments, and services. Finally, challenges to developing new treatments remain a concern. Finding ways to reduce the issues regarding the enrollment, design, and replication of compelling clinical trials is paramount.

### 3.3. The Societal Impact

The impact of RDs can be diverse and affect different areas of the lives of patients and their families. In this section, we will provide an overview of how patients, their families, and their caregivers are affected in the dimensions of professional, educational, and social life, as well as psychological and emotional wellbeing. While the societal impact of RDs is still largely limited in literature, siloed between disciplines [8], it constitutes an essential piece to understanding the full scope of the multidimensional impact (burden) of RDs.

#### 3.3.1. General Overview of the Societal Impact

Despite the variety of clinical manifestations of RDs, patients face a myriad of common challenges associated with difficulties encountered during their journeys, including the direct suffering that derives from the clinical, physical, and emotional impacts of their diseases [107]. The significant impact of RDs on the mental health and personal wellbeing of RD patients can manifest itself in different ways, with patients predominantly presenting behavioral problems, depression, and violent outbursts, among others [108]. People living with an RD might have difficulties with daily activities, which might hinder the possibility of having an active social [109] and/or professional life [14]. This can lead many RD patients to experience strong feelings of isolation, which are also often fueled by family tensions [108]. The impact of RDs on the capacity of patients working long hours can be a significant barrier to their employment [14] and ability to participate in education. 

The families of patients living with RDs also encounter challenges in their professional, social, and family lives. In the absence of policies and regulatory frameworks favorable to affordable access to RD treatment and care, RD patients must often rely on their family network to provide care and help cover out-of-pocket costs related to their conditions. Providing care for RD patients is time intensive, forcing many caregivers to reduce or stop their professional activity, putting households under financial strain [110]. 

#### 3.3.2. Regional Overview of the Societal Impact

In North America, evidence from the United States shows that RD patients suffer from worse anxiety, depression, fatigue, pain, and ability to participate in society than most of the general population and people with common chronic conditions [76]. The significant impact that common chronic conditions have on the quality of life of patients is widely discussed in the literature [111]. Evidence from the United States indicated that the higher impact on quality of life when compared to those with common chronic diseases is not inherent to the pathology of the disease itself, but due to challenges associated with insufficient funding and infrastructure for research, treatment, and psychosocial support that RD patients face [76].

Caregivers’ mental and personal wellbeing is also compromised. A quantitative survey of RD caregivers in the United States found that more than half of respondents spend 40 h or more a week providing care, and in most cases, full-time care. Moreover, it was found that only around 18% spend less than nine hours per week on these activities and that RD caregivers spend more time on average providing care than general caregivers (37 vs. 25 h for caregivers of adults; 53 vs. 30 for caregivers of children). [12]. Caregiving activities were also found to interfere with professional life. Nearly 91% of caregivers reported having gone late to work or left early to provide care [12]. According to the same survey, one in five caregivers also reported experiencing isolation from their family network [12], and in Canada, caregivers have reported suffering from a sense of isolation and financial vulnerability. According to a survey, almost 80% of RD caregivers suffer from mental health issues as a result of their caregiving responsibilities, and 63% have taken on debt to cover the associated cost of their caregiving responsibilities [15].

In Latin American countries, the impact of RDs on society is exacerbated by the lack of specific policies and regulatory frameworks. The reduced availability and extremely high prices of orphan drugs have led to an increasing trend in the judicialization of healthcare services [112,113]. Patients and their caregivers often face long and devastating legal battles with health authorities and national insurance providers to obtain reimbursement for the treatment of RDs [113]. Moreover, although there is increasing awareness of RDs in the region, pushing several countries to approve laws that ensure access to diagnosis, treatment, and care, many lack the resources and capacity to fully operationalize dispositions and provide adequate care [30,106,114]. 

In Europe, a survey conducted in 42 countries confirms how much RDs impact the wellbeing of patients and caregivers [14]. According to the results, more than 70% of people living with an RD have difficulties with daily activities and tasks such as household chores, preparing meals, and shopping. Forty-two percent of caregivers spend more than two hours per day on illness-related tasks, such as hygiene and treatment administration, and thirty percent spend more than six hours per day on illness-related tasks. This number increases from 30 to 47% for caregivers attending severely affected individuals. Seventy percent of patients and caregivers had to reduce or stop their professional activity due to the disease, and 21% were found to be absent from work more than 90 days per year. For comparison, another study found that the number of individuals who were absent from work in 2020 due to their own illness or disability at the European level was 2.9% of employed people [115].

Like in Latin America, RD patients from Asia-Pacific countries must also rely on their family network to provide care and cover the out-of-pocket costs associated with their diseases. A study in China revealed that 83% of RD patients had no disability certificates (which are issued by the government), a necessary requirement to access benefits and protection provided by the government. The low number of patients with disability certificates is not only an issue for RD patients but people with disabilities in general, which may be associated with the bureaucracy of the process [116], stigma, and discrimination [117]. Not surprisingly, 69.6% of RD patients relied on the income of family members [118]. Similarly, RD patients in India often report absenteeism from work/school due to their diseases, leading to higher rates of school dropouts and unemployment when compared to the general population [54]. Moreover, in Australia, 40% of RD caregivers are actively treated for depression and anxiety [17,67].

Formal studies on the societal impact of RDs in Africa and the Middle East are not available. The lack of research and resources to properly diagnose and monitor RDs in Africa and the Middle East makes it impossible to assess their impact on society. This is particularly concerning, as common practices in these regions are proved to be strong contributing factors in higher levels of congenital and genetic disorders, namely advanced maternal and paternal age and high consanguinity.

#### 3.3.3. Gaps, Barriers, and Challenges 

There is limited recognition and awareness of the full impact of RDs on patients and their families from a societal perspective. Equity, social justice, and social protection mechanisms are fundamental pillars of public health, yet RD patients and their families often feel socially and culturally excluded. There is a global need to continue addressing the root causes of discrimination and stigma of people living with RDs [119,120]. Ensuring that decision makers and the public at large recognize and are aware of the full social and economic impact of RDs and the needs of these populations is essential, especially in light of persistent disparities across and within countries. The absence of evidence limits the possibilities countries have to implement comprehensive measures and policies to reduce the social impact RDs have on patients and their caregivers. 

Efforts of this nature should consider the full range of experiences that patients and caregivers endure along the care journey, as well as the social and economic implications to their livelihoods and society. Return on investment studies that can capture the full scope of impact are highly valuable for policy change, repositioning that testing, treatment, and care for RDs constitute an “investment” rather than an “expenditure.” However, conducting this kind of study is particularly challenging in resource-constrained settings. 

Caregivers and families are highly impacted by RDs at various levels, regarding their health, work-life balance, education, and social life. Providing care for RD patients is difficult and time-consuming. Patients and their families often miss significant social interactions and face challenges holding jobs and finishing educational pathways. This further puts patients and families in a vulnerable economic position. The loss of productivity also means additional economic losses to the country. The lack of comprehensive services that can accommodate the full range of patients’ and their families’ needs generates feelings of frustration, which could eventually lead to self-excluding. The substantial impact RDs place on patients, caregivers, and countries urges the prioritization of RDs in national and international public health agendas.

### 3.4. Disease Management

Disease management refers to the use of strategies and programs to manage patients affected by specific diseases in identifying, treating, and monitoring their progress to adequately address their health needs. To complement what has already been presented regarding the patient journey, in this section, we will focus on the challenges faced by patients regarding the adequate management of their conditions. 

#### 3.4.1. Global Overview of Disease Management

A global overview of orphan drug policies (encompassing acts, decrees, guidelines, ordinances, and policies) found that 92 countries/territories (46%) have legislation, regulations, or policies that facilitate patient access to orphan drugs. However, significant geographic disparities were identified. Only 19% of the 31 low-income countries/territories included in the sample had in place some kind of policy to facilitate access to drugs for RDs. The main policy gaps identified include price regulation, incentives that encourage market availability, and incentives that encourage research and development [121]. 

According to our findings, challenges regarding RD management can be organized into four groups, given their consequences: lack of public awareness, late diagnosis, issues with access, and lack of coverage. Regarding the first, in a general sense, we found that healthcare providers, decision makers, and the population at large have limited knowledge and awareness of RDs. Not only is information on RDs limited, but there are also few opportunities to raise awareness and educate key stakeholders. This is worsened by the lack of opportunities for patients’ voices to be heard and respected during policy decision-making [122,123]. 

From a disease management perspective, challenges regarding diagnosis derive from the lack of comprehensive training of healthcare professionals on RDs, limited capacity to provide diagnostic services (the lack of or limited affordable genome sequencing, laboratory capacity, and specialists), and discrepancies regarding availability of screening programs between countries [56,67,76,78,124]. Challenges regarding access to treatment derive from similar sources. Especially in rural settings, access to specialist diagnosis and treatment centers for RDs is sparse. National policies, healthcare budgets, health insurance, and reimbursement systems also influence patient access to diagnosis, treatment, and care, with insurance being a common gatekeeper to receiving appropriate treatment in many contexts [58,78,81,124,125]. 

Lastly, regarding coverage, orphan drugs are often very expensive, and various reimbursement and pricing systems across countries create disparities in patient access to adequate treatment. Furthermore, the prioritization of other public health issues might, in some contexts, lead to insufficient resources being assigned to RDs [67,78,81,85,126,127,128]. Finding a balance of attending to public health priorities without disregarding the needs of patients living with RDs is a challenging task.

#### 3.4.2. Regional Overview of Disease Management

As mentioned earlier, in North America, despite growing investment in RDs, patients typically live without a diagnosis longer than in more common diseases and must endure many physician visits to receive a confirmed diagnosis [76]. In 2019, the National Organization for Rare Disorders conducted a survey with RD patients in the United States and found that only 29% of RD patients had been granted access to treatments that were not approved by the FDA for their medical conditions, 61% had been denied or faced delays accessing treatments that required pre-approval from an insurance company, and 18% had been denied referral to a specialist [81]. Off-label prescribing is legal and common in the United States, with many RD patients relying on it [81]. However, insurers usually require more than a doctor’s prescription for off-label coverage [129], such as inclusion in reputable and authoritative reference lists [130]. 

While insurance approval is the main gatekeeper to accessing treatment, the business model of pharmaceutical companies contributes to the increasing availability and indication of orphan drugs. Pharmaceutical companies are driven towards orphan indications due to a market push, characterized by saturation in the market of broad, common indications, and a technology pull that attracts them to RDs. Not only do RDs generally have high clinical unmet needs, making it easier to obtain a large market share and get reimbursement for high prices, but progress in technology, especially in genomics, gene therapy, and antisense therapy, has made it possible to identify and treat RDs in a way that was not possible some years ago [131]. In the United States, the total number of approved indications and drugs, respectively, reached 838 and 564 in 2019. Notably, the number of approved orphan drug indications is growing even faster than the number of drugs, as 25% of those have multiple indications [132]. 

In Latin American countries, there are few discussions about symptoms, complications, and possible cures for RDs [97]. Beyond the lack of public awareness, patients also face challenges when seeking a diagnosis, such as genome sequencing and laboratory capacity being generally limited, with almost all specialists in RD diagnosis and treatment centers based in urban areas. Furthermore, orphan drugs are often not reimbursed within public health systems, which disproportionally impacts access for low-income patients [30,127]. Services are organized for and cater to higher-income families who can afford the out-of-pocket payments and private health insurance costs required for the diagnosis and treatment of RDs. In Chile, for example, a study reports that families that can pay out-of-pocket have their next-generation sequencing-based tests sent to international clinical laboratories [124]. Therefore, the quality of RD care and treatment in Latin American countries is extensively dependent on the socioeconomic status of the patient affected (a situation that also applies to the population in general) [133,134].

In European countries, there are different screening practices across borders. Newborn screening (NBS) is particularly important to people living with RDs and their families, as the onset of most of these conditions occurs during childhood. In a general sense, the number of diseases screened for as part of NBS programs varies greatly between European countries [135]. A systematic review found these range from two to thirty-five across ten countries [85]. Regarding reimbursement, evidence suggests that systems with decentralized health decision-making might experience significant geographic inequities within their territories in access to treatment for RD patients, as well as the population in general [136]. This is the case in Spain, where an analysis conducted in 2017 of non-oncology orphan drugs revealed that 69% of the drugs licensed by the European Medicines Agency between 2012 and 2016 had not received a pricing and reimbursement decision, 29% had not sought a national code, and 40% were still undergoing assessment [137]. Not surprisingly, a survey conducted the same year found that almost half of RD patients had to travel outside of their region to access treatment and 17% lacked care because they were unable to travel [138]. Patient access to orphan drugs and healthcare services also differ greatly between countries. This is particularly evident considering variations in national policies, healthcare budgets, types of healthcare and health insurance systems, reimbursement systems, and patient co-payment rules [85,125].

In the Asia-Pacific region, evidence is indicative of low medical awareness and a lack of momentum in acknowledging and addressing the unmet needs of patients. This is also exacerbated by the paternalistic health systems in the region, in which patients’ voices are seldom heard or respected [122]. A report from 2020 found that 14% of healthcare professionals did not feel confident diagnosing and managing RDs, nor have they ever encountered a patient with an RD in their careers [67]. Training and learning opportunities are limited. In India, for example, a study found that training and education opportunities for medical professionals mainly focus on common diseases [53]. Furthermore, countries in the region must also deal with limited RD healthcare capacity, possessing limited genome sequencing laboratories [78], and for those who do get diagnosed, treatment is usually pricy, as orphan drugs are generally not reimbursed within public health systems [67]. 

In Africa and the Middle East, there is limited knowledge and awareness of RDs among healthcare providers. One of the leading reasons has to do with the lack of availability of training opportunities on RDs for healthcare professionals [123]. A report on the care of RDs in Africa highlights the limited availability of genetic testing [123], while another study also identifies the lack of RD systematic registries [128]. In fact, the ratio of geneticists per population in Africa is much below the recommendation by the WHO [56]. Countries in Africa and the Middle East also have limited infrastructure for the diagnosis and treatment of RDs, with most centers available only in urban areas. In Africa, because countries are severely affected by communicable diseases such as HIV and tuberculosis, and non-communicable diseases as well, much of the funding and prioritization goes to these diseases, leaving minimal resources for other conditions such as RDs [128].

#### 3.4.3. Gaps, Barriers, and Challenges

One of the essential components to ensure quality treatment and care for patients with RDs is to have a supportive and well-developed infrastructure and knowledge; however, both are not widely available, reducing the odds of patients having basic access to diagnosis. The treatment and care of RD patients carry a substantial economic burden [20] and controlling these prices to provide adequate disease management and ensure the financial sustainability of health systems is challenging.

The absence of clinical guidelines for RDs might increase the chances of misdiagnosing patients, as well as limit the capacity of healthcare providers in defining the best treatment to manage each disease. As both infrastructure and healthcare professionals with adequate knowledge and experience are usually concentrated in a few centers, mostly in urban areas, disease management efforts at local and regional levels are undermined. Challenges in treatment are exacerbated by the fact that patients with RDs often require care from multiple specialties. Providing care in this context requires highly interoperational services that can help providers coordinate and manage patients effectively. Sadly, these kinds of systems are absent in most countries [30]. 

It is important to acknowledge that local needs are as important as global reach. Priorities in disease management depend on each individual context. For example, in countries that lack RD policies, screening services, and diagnostic and treatment facilities, national clinical RD guidelines are particularly relevant to ensure adequate and quality care [9]. Finally, while the lack of reimbursement and pricing models challenges access to treatment, it also hampers the interest of pharmaceutical companies to continue to invest in research and development for RDs [67]. Given that only 5% of RDs have a treatment available, leaving most patients with no treatment options [9,53], ensuring a supportive environment for RD health innovation should be a priority.

### 3.5. The Policy Landscape

There is a clear need to prioritize RDs in the public health agenda given the socioeconomic impact they have on patients and society and the complex challenges associated with the patient journey and disease management. Given that treatment options for many RDs involve high-cost orphan drugs, the implementation and application of appropriate public policies is paramount to give rise to suitable solutions.

#### 3.5.1. Global Plans and Strategies on RDs

In a general sense, RDs have only recently been recognized by leading international organizations as a topic of concern. RDs were featured for the first time on the agenda of the WHO during the 72nd World Health Assembly, which took place in May 2019. At this event, RD case studies were used to highlight how transformational digital technologies can contribute to achieving the universal health coverage principle of leaving no one behind [139].

In December 2019, RDI, the global alliance of persons living with an RD, signed a landmark agreement for collaboration with the WHO, including the development of a Collaborative Global Network for Rare Diseases and an international Operational Description of Rare Diseases [140]. The Collaborative Global Network is an initiative to develop a global, person-centered network of care and expertise for RD patients with the mission to develop local capacities, raise awareness amongst stakeholders, break down barriers to care, connect multi-disciplined expert centers and teams, reinforce existing expertise, and encourage the development of a global learning health system through the sharing of information and data. The components of the network include National Hubs, Regional Hubs, and a Global Network [3].

Similarly, in 2019, the United Nations (UN) demonstrated a commitment for the first time to reduce the burden of RDs as part of achieving universal health coverage [141]. By 2021, the UN adopted a resolution on RDs (Resolution A/RES/76/132: Addressing the Challenges of Persons Living with a Rare Disease and their Families) recognizing the needs and challenges of RD patients and their families, including the issue of access to medicines [142]. Regarding this topic, the resolution calls upon Member States to strengthen and implement national measures that address the physical and mental health needs of RD patients and their families to realize their human rights.

These global plans and strategies on RD, although recent, hold great potential to promote and/or accelerate national action for the development/implementation of appropriate healthcare policies, strategies, plans, and regulatory frameworks.

#### 3.5.2. Regional RD Policy Landscape

North American countries have well-established and comprehensive RD policies and regulatory frameworks. In the United States, the Orphan Drug Act from 1983 and the Rare Diseases Act from 2002 have changed the RD landscape [73], providing incentives such as market-exclusivity deals, tax credits, clinical research subsidies, protocol assistance, FDA registration fee exceptions, and increased federal funding for the development of RDs treatments [30]. Similarly, in Canada, the Health Canada Special Access Program and the Orphan Drug Framework provide access to several orphan drugs for RD patients, as well as facilitate research [30]. Currently, the government is also working towards launching the national strategy for RDs drugs in 2022 [143]. 

RD policies across Latin America vary greatly, with some countries (e.g., Argentina, Brazil, Colombia, Ecuador, Panama) exhibiting comprehensive RD healthcare policies, such as the presence of healthcare plans with national centers and facilities to support disease management and guarantee access to health services. A second group of countries (e.g., Guatemala, Mexico, Peru, Uruguay) have only basic protective laws that do not necessarily guarantee funding or financial protection for RD patients, nor do they regulate access to treatments. Finally, a third group of countries (e.g., Venezuela and Cuba) have yet to codify specific laws to protect, treat, or research RDs. In these countries, treatments are neither financed nor protected by the government. Nonetheless, regulatory frameworks in the region are relatively new [28,30,73,106,124,144]. 

Unlike other regions, European countries have a common comprehensive body of RD-related policies, mechanisms, and regulations. Regarding this latest, the EU has a multinational legislation for orphan drugs called the Orphan Medicinal Product Regulation (EC) No. 141/2000, which provides a series of incentives for the development of RDs medicines [145]. In 2008, the EU also launched the European Project for Rare Disease National Plan (EUROPLAN), aiming to facilitate the development of national plans in the region, becoming an instrumental tool to stimulate debate around RD policy across Member States. The Filières de Santé Maladies Rares’ (FSMR), established in 2014 in France, aims to connect centers with expertise in the same broad disease areas, and represents an important precedent for the European Reference Networks (ERNs) [146]. The ERNs are virtual networks connecting RD healthcare professionals, involving more than 900 highly specialized healthcare units from over 300 hospitals in 26 EU countries, allowing professionals to discuss patients’ diagnoses and care [147]. The EU Screen4care project was launched in October 2021 by an international public–private consortium of 35 partners. The five-year project aims to significantly shorten the time required for RD diagnosis and efficient intervention by utilizing genetic NBS and advanced analysis methods such as machine learning [148]. Finally, the Rare 2030 Foresight Study gathered the input of a large group of stakeholders to propose recommendations to improve policies and better the future of RD patients in Europe, culminating in a presentation to the EU Parliament in February 2021 [149]. 

Besides regional mechanisms and frameworks, countries in Europe have their own RD strategies and plans [78]. For example, the United Kingdom has a Strategy for Rare Diseases (2013), a Rare Diseases Framework (2021), and the England Rare Diseases Action Plan (2022) [150]. Countries have also launched national initiatives for RDs research and development, such as incentives to support research programs in the Netherlands, as stipulated in the National Plan for Rare Diseases in 2013 [151]. Non-EU countries also have national regulations to ensure coverage of RD drugs. In Russia, for example, there is a special program that provides financing for 12 high-cost diseases at the federal level [152].

The European Commission has been heavily engaged in the development of RD policies. One example is the support provided during the revision of the International Classification of Diseases (ICD), ensuring that RDs are considered and aligned to the coding used within Orphanet [153]. The use of a common coding system can facilitate case reporting, death reporting, and, in some cases, decision-making regarding reimbursement and resource allocation. The 11th revision of the ICD came into effect in 2022, and it includes about 5500 RDs and their synonyms [154].

Several of the major economies in the Asia-Pacific region have legislation on incentives focused on RDs research and development. This is, for example, the case in Australia, Japan, and Taiwan, where measures provide financial and marketing incentives to develop and produce RD medicines. In Taiwan, the Rare Disease and Orphan Drug Act (2000) provides grants, fast-track approval, protocol assistance, and market exclusivity [30]. Similarly in Japan, the revised Orphan Drug Regulation (1993) and the Revision of Measures to Combat Intractable Diseases (2013) provide regulatory fee waivers, research grants, tax credits, and reductions, tools to reimburse medical costs, and funding to encourage research and orphan drug development. In 2015, the Pharmaceuticals and Medical Devices Agency and the Ministry of Health, Labor, and Welfare of Japan instituted a designation system, Sakigake, that provides support for the development of innovative new drugs through financial incentives, market exclusivity, and priority review; it is expected that this system will help resolve the issue of delayed approvals in the future [100,155]. 

Some countries in the region have also developed and implemented RD national policies and programs to increase the diagnosis and care of RD patients, including screening programs. This is, for example, the case in Thailand, Italy, the United States, and Singapore. In Thailand, the government introduced a Rare Disease Policy and treatment for 24 RDs (inborn metabolic disorders) into the benefits package of Universal Coverage in 2020 [2] and expanded the newborn screening program to include 40 RDs in 2022 [2,156]. In Italy, newborn screening has been a preventive, mandatory, and free health activity since 1992, initially covering only three diseases but eventually expanding, in 2016, to include 40 additional hereditary metabolic diseases throughout the whole national territory (Law 167/2016) [157]. In the United States, the Recommended Uniform Screening Panel identifies a list of 36 core conditions and 26 secondary conditions recommended for screening at newborn screening programs across states [158]. In Singapore, the Orphan Drug Act (1991) provides more than 25 metabolic-related screening tests under the National Expanded Newborn Screening Programme [80]. In Japan, the Initiative on Rare and Undiagnosed Diseases (IRUD) was established as a nationwide program to provide an accurate diagnosis, discover causes, and ultimately provide cures for rare and undiagnosed diseases. The program has since achieved important results regarding accurate diagnosis and identification of relevant genes [100,159,160]. Regarding care, policies to provide subsidies and increase knowledge and awareness have also been implemented. For example, in Australia, the Life Saving Drugs Program subsidizes expensive and life-saving drugs for patients, and the National Strategic Action Plan for Rare Diseases (2020) seeks to increase awareness and education, care and support, and research and data [86].

African and Middle Eastern countries broadly lack RD health policies. However, some private initiatives can be identified. To name a few, in South Africa, the North-West University’s Centre for Human Metabolomics is in the process of establishing the first RD biobank in the African continent, with the main focus on collecting samples and information on rare congenital disorders [56,161]. In Lebanon, the American University in Beirut, since 2009, has offered neurological and genetic diagnostic and treatment services through their Neurogenetics Centre of Excellence, as well as state-of-the-art research facilities. In Saudi Arabia, significant investment has been made in the education sector with the opening of numerous universities focused on biotechnology [30,73].

#### 3.5.3. Lessons Learned from Regional Case Studies

In North America, among policies that had a positive consequence on RDs is the Newborn Screening Saves Lives Act, passed by Congress in 2008. It facilitated the expansion of NBS panels in the USA by improving the assessment, coordination, and treatment of infants by educating and training laboratory personnel in screening programs and technologies [162]. Similarly, the Orphan Drug Act, signed in 1983, created tax incentives and credits, financial support for research and development, and accelerated approval [162], encouraging interest and accelerating research on RDs, allowing patients to have access to treatment [163]. While the Orphan Drug Act has resulted in the approval of over 650 orphan drugs [164], it has also been found to be associated with some negative consequences. The extended market exclusivity of seven years guaranteed by the Act has been associated with unacceptably high drug prices, both for newly developed drugs and even for drugs that were previously widely available [165]. 

In Latin America, initiatives such as tax benefits have sought to facilitate access to high-cost orphan drugs. For example, in Chile, Law 20.850 funds medications, medical devices, or nutrition for 29 health conditions, including 14 RDs, through general taxes [124], enhancing coverage and access to treatment for RD patients [106]. There are also positive consequences in the region from measures to provide support to healthcare professionals, leading to higher numbers and better accuracy in the diagnosis of RDs. One example is the Information Service on Metabolic Diseases (SIEM) from Brazil, which provides specialized information and guidance to help physicians and health professionals involved in the diagnosis and management of patients presenting any type of inherited metabolic disease [166]. While there are success stories, implementation continues to be a challenge in many contexts. In Argentina, for example, Decree 794 to implement the National Rare Disease Policy created regulatory ambiguity by leaving most of the specific mandates unregulated, slowing down efforts to address RDs comprehensively in the country [106]. Moreover, in Chile, there are concerns that the program established by Law 20.850 is insufficient to resolve patients’ unmet medical needs and is unsustainable over time [106].

As expressed earlier, in Europe, the Regulation on Orphan Medicinal Products brought positive consequences in terms of access to treatment for RD patients. This regulation introduced a comprehensive set of incentives, including market exclusivity for ten years, in addition to regular protection [167]. Since then, the number of authorized products increased from eight to 164 for approximately 90 RDs. Research and development also improved, with the number of RD clinical trials increasing by 88%, and 25 European countries adopted at least one national RD plan by 2020; although, a study found that only 13 of these could be deemed ”active” at the time [168]. Market exclusivity, however, can permanently deter generic products from competing in the region [167]. Since companies cannot a priori know if and when the generic competition will emerge, they will likely price products at a premium upon market entry. The absence of competition can lead to retaining the premium price indefinitely [167].

In the Asia Pacific region, some countries have successfully managed to include socioeconomic factors during reimbursement decision-making on RDs drugs. For example, in Thailand, drugs for hemophilia (covered since 2007) and for Type 1 Gaucher disease (covered since 2013) were still rendered a positive choice despite the unattractive cost-effectiveness ratio following multidimensional socioeconomic analysis [80,169]. By implementing policies and legislation to provide subsidies, many countries in the region have also managed to increase the protection of RD patients. The Rare Disease Act of the Philippines (2015) categorized patients with RDs as “persons with disability,” thus allowing patients to access statutory benefits, including discounts on healthcare services and medicines [80]. In Taiwan, the Rare Disease and Orphan Act (2000) introduced financial subsidies and exclusive marketing measures allowing RD patients to get between 70 and 100% reimbursement on orphan drugs (according to their income level) [80]. However, in other cases, subsidies fail to reach the RD community at large. In Malaysia, for example, subsidy requirements for patients excluded large portions of the population, covering only selective numbers of therapies and treatments [80]. 

The lack of RD policies in Africa and the Middle East makes it difficult to discuss lessons learned from experiences in the countries. 

#### 3.5.4. Gaps, Barriers, and Challenges

International organizations and institutions have recently started to focus on RDs. To this day, there is no global overarching strategy or plan for RDs. A globally coordinated RD strategy could avoid duplicating efforts and improve the sharing of knowledge and expertise across countries. A global approach would also be beneficial in reducing the gaps observed between and within regions. 

Only Europe has a regional RD approach. Most countries in each region have individual RD strategies, policies, and regulations. There is a great risk that the absence of a common approach might perpetuate, given that the visibility of the RD community might be limited in several countries and contexts (the RD community is a heterogeneous group scattered across countries/continents). Progress is also challenged by the limited capacity and resources of PAGs. The RD patient advocacy community is often under-resourced to effectively shape discussions, educate policymakers, and drive the successful implementation of RDs programs. 

### 3.6. Research and Development

Evidence is fundamental for public health decision-making, as well as for the development of health innovations and technology. In this section, we will introduce the issues and challenges of developing evidence across different types of studies: clinical trials, genomic studies, and cost-of-illness studies. We also include a reflection on the pivotal role that patients’ registries play in the development of evidence. 

Clinical trials are used during the process of drug development to evaluate their effects on health outcomes. These studies employ statistical analysis of the information collected to allow clinical researchers to form reasonable inferences and sound decisions. Human genomic studies are a new and rapidly evolving branch of science to study human genetic material. Applied to public health, genomic studies bring benefits in refining diagnosis and guiding therapeutic approaches for diseases such as cancer, heart diseases, and genetic conditions, providing new approaches to preventing and managing many intractable/hard-to-treat diseases. Cost-of-illness studies are essential for the evaluation process in healthcare, as the measurement and comparison of the economic burden of diseases to society helps decision makers set up and prioritize healthcare policies and interventions. Economic evaluation is a fundamental aspect of HTA with the goal of addressing the increasing healthcare needs of society in the context of limited resources, in terms such as value for money or affordability. The economic evaluation of drugs encompasses the difficult challenge of finding a balance between providing high-quality, innovative care, selecting appropriate measures for funding, and the financial sustainability of countries. Patient registries can provide important information about the course of diseases, treatment effectiveness and outcomes, quality of life, care patterns, and monitoring of patients over time. To identify, follow-up with, and analyze, information on these patients is relevant to conduct the types of studies discussed in this section and to generate valuable evidence for disease management overall.

#### 3.6.1. Issues and Challenges Developing Evidence through Clinical Trials

Researchers face many barriers to designing and conducting clinical trials to develop new RDs medicines. Perhaps the most obvious challenges derive from the nature of the RD population. This population is a small, heterogeneous, and widely dispersed group, complicating the enrollment. Given the rarity of these diseases, there is a small pool of available participants for clinical trials [170], a situation that is worsened by the frequent use of rigid inclusion and exclusion criteria [170]. For example, clinical trials which require treatment-naïve participants might have difficulties enrolling participants as only a small proportion of patients fulfill this condition [171]. This also has implications in the power attributed to findings by decision makers, which may limit reimbursement and therefore access to treatments. While multicenter studies can help enroll more patients, it further complicates the study design, and even, when possible, the presence of few study sites across countries complicates gathering large amounts of high-quality data that is sufficient to reach statistically significant values. 

The heterogeneity of RD patients, regarding their clinical presentation and histories, including age, disease progression, and disease severity, renders it difficult to reach a consensus on clinical outcome measures and define endpoints of analysis. These measures are necessary to evaluate the effectiveness of new medicines. Furthermore, since over half of the population affected by RDs are children, special ethical considerations limit the possibility to enroll this population in clinical trials, which can slow down the development of new therapies. It is important to standardize RD research approach and technology to improve the chances to compare data [172]. 

Finally, clinical trials are also made challenging by limited funding. Pharmaceutical companies might be discouraged to continue to conduct and invest in this kind of study due to at least two reasons: one, clinical trials can be expensive, time-consuming, and risky, and two, clinical trials need to comply with regulatory requirements regarding safety, efficacy, and quality [173]. 

#### 3.6.2. Issues and Challenges Developing Evidence through Human Genomic Studies

Since most RDs have a genetic origin [174,175], the study of human genes and chromosomes is vital to produce reliable evidence regarding RDs. However, currently, there are two main limitations associated with the evidence produced through human genomic studies. The first one has to do with the under-representation of ethnically diverse populations in these studies. This has important implications for the interpretability of genomic variants and diagnostic assessments. Genome sequencing is often used in the diagnosis of RDs; thus, reference genomes from more ethnically diverse populations are needed for reliable interpretation of results of minority populations [176]. Yet, a study found that only 22% of individuals in genome-wide association studies were of non-European ancestry. People of African and Latin American descent and Indigenous people combined represented less than 4% of participants [176]. Recent initiatives are trying to amend this underrepresentation by undertaking genomic studies in non-European/North American countries, such as Brazil [177], Thailand [178], and South Africa [179]. 

Nonetheless, with new initiatives being undertaken in Africa, concerns regarding the ethical use and management of genetic samples have increased. Some of these concerns include genomics literacy, good governance for genomics and biobanking, and protection of patients, the public, and data [161].

#### 3.6.3. Issues and Challenges Developing Evidence through Cost-of-Illness Studies

Cost-of-illness studies are often limited by the lack of primary and/or aggregated data, which prevents a reliable estimation of the economic burden of disease, and even when available, the heterogeneity of these kinds of studies limits the possibility of comparing results and extrapolating findings. In fact, most evidence on the burden of disease depicts the reality of only a few (and better off) countries. Most cost-of-illness studies have been conducted in developed countries, such as the United States, Canada, and European countries [62].

There is also a lack of consensus on a clear methodology to perform cost-of-illness studies and particularly on the inclusion of indirect costs aside from the loss of productivity. Most studies include only costs incurred by patients, such as those associated with out-of-pocket expenses or informal care. This comes to exemplify that studies often do not distinguish payers in their methodology. Without a clear and realistic picture of the full scope of the burden of disease, decisions regarding the reimbursement of drugs will remain limited [62].

#### 3.6.4. The Need for Patient Registries

Patient registries constitute a key instrument for increasing knowledge of RDs, supporting fundamental clinical and epidemiological research and clinical trials, post-marketing surveillance of orphan drugs and treatments used off-label, and health and social services planning (playing a pivotal role in healthcare organization) [180,181,182]. Furthermore, the consistent longitudinal collection of patient data facilitates the creation of standards of care and dramatically improves patient outcomes and life expectancy even in the absence of new therapies [182]. Moreover, in combination with the presence of patient organizations, registries also increase the likelihood of treatment development [183]. 

According to a joint effort by the EURORDIS, the National Organization for Rare Disorders (NORD), and the Canadian Organization for Rare Disorders (CORD), there are ten principles RD patient registries should follow [182]:

- Patient registries should be recognized as a global priority in the field of RDs.
- RD patient registries should encompass the widest geographic scope possible.
- RD patient registries should be centered on a disease or group of diseases rather than a therapeutic intervention.
- Interoperability and harmonization between RD patient registries should be consistently pursued.
- A minimum set of Common Data Elements should be consistently used in all RD patient registries.
- RD patient registries data should be linked with corresponding biobank data.
- RD patient registries should include data directly reported by patients along with data reported by healthcare professionals.
- Public–private partnerships should be encouraged to ensure the sustainability of RD patient registries.
- Patients should be equally involved with other stakeholders in the governance of RD patient registries.
- RD patient registries should serve as key instruments to build and empower patient communities.

## 4. Recommendation to Improve Access to RDs Diagnoses and Treatments

Based on overviewed evidence, 11 global priority areas for intervention were identified, namely:

1. Ensuring research and development of essential evidence.
2. Encouraging investment in research and development for RDs.
3. Building equitable access to diagnosis, treatments, and care.
4. Building capacity and awareness of healthcare workers (HCWs).
5. Improving healthcare system and services for RD patients.
6. Standardizing clinical guidelines.
7. Investing in capacity-building.
8. Ensuring patients’ participation in decision-making.
9. Strengthening stewardship and accountability.
10. Building public awareness.
11. Supporting the development of diagnostics.

For each priority, between two and eight recommendations are provided. The recommendations are derived from the discussion undertaken by the experts on the findings of this review and seek to provide a set of actionable solutions to the challenges and barriers to improving access to RD diagnosis and treatment around the world. The recommendations are meant to serve as an umbrella that country stakeholders and decision makers can choose from and use according to their needs, priorities, and resources.

1. Ensuring research and development of essential evidence
  (a) Improve national surveillance mechanisms and registries to gather sufficient information to support research, health and social services planning, and policy-shaping.
  (b) Improve monitoring and evaluation of data.
  (c) Improve data standardization, centralizing, and sharing to develop mechanisms to support the identification of RDs.
  (d) Collectively develop new ways of defining and measuring value, reaching an international agreement on a consistent multidimensional socioeconomic measurement to assess the impact that captures the full scope of benefits for patients, their families, and the health system.
  (e) Reach a consensus on clinical outcome measures and defined endpoints of RDs treatments.
  (f) Implement/strengthen mechanisms to broaden the research sample by including global data and resource-limited countries in early research.
  (g) Engage patients in the entire product development lifecycle, including priority-setting, design, and execution of clinical trials, value-assessing, and access decision-making.
  (h) Global coordination for the diagnosis of patients through innovative technologies.
2. Encouraging investment in research and development for RDs
  (a) Provide incentives for manufacturers and researchers to invest in the development of orphan drugs and innovations for more effective RD treatment.
  (b) Ensure governments understand that investment in research and development for RDs is an investment of high return for patients’ health, quality of life, and wellbeing.
  (c) Ensure that governments, manufacturers, researchers, venture capitalists, and PAGs promote and invest in RDs research and development.
  (d) Promote the international community to develop and disseminate a global overarching strategy or plan for RDs.
  (e) Develop policies and procedures to lower the cost of approvals of new RDs drugs and treatments.
3. Building equitable access to diagnosis, treatments, and care
  (a) Ensure universal access to health services and treatment (essential medicines as well as advanced therapies such as biotherapeutic products) are in line with the UN Sustainable Development Goals (SDGs) and Universal Health Coverage.
  (b) Prioritize RD as a group in national health systems (using criteria beyond frequency) and improve reimbursement and regulatory processes to increase affordability.
  (c) Implement and/or strengthen tools and mechanisms to control costs of treatment, including for RDs, ensuring high quality of care and sustainability of health systems.
  (d) Identify and implement alternate funding mechanisms to improve reimbursement of innovative treatments to combat access barriers.
  (e) Ensure HTA and reimbursement decision of RDs drugs and technologies is performed based on a multidimensional socioeconomic analysis and the nature of the RD.
  (f) Include psychosocial services for patients and caregivers as part of the standard of care for RDs.
  (g) Establish disease-specific and non-disease-specific centers of excellence for ultra-RDs across the globe according to the European Reference Networks example.
  (h) Ensure comprehensive care plans and policies are in place (such as adequate referral mechanisms/systems) to close the gap in access between rural and urban settings.
4. Building capacity and awareness of healthcare workers (HCWs)
  (a) Increase capacity/knowledge of HCWs on patient experience, symptoms, and impact of RDs; types of care and treatment; and disease progression through regular training opportunities, including through employee training and development programs.
  (b) Include education on RD as part of the healthcare education curriculum.
  (c) Consult with, and seek input from, medical societies to incorporate their expertise into RD medical education programs.
  (d) Implement training programs on RDs for HCWs in primary healthcare centers.
5. Improving the healthcare system and services for RD patients
  (a) Ensure early access to genetic screening, and referral consultation network, including specialist care, integrated services, infrastructure, and human resources.
  (b) Governments across the globe should implement a people-centered model of care that is respectful of, and responsive to, the preferences, needs, and values of patients and that provides emotional support, physical comfort, information and communication, continuity and transition, care access and coordination, and the involvement of patients’ families and caregivers.
  (c) Implement institutional information tools, such as websites, helplines, etc. to guarantee that all RD patients have access to critical information and support.
  (d) Government should invest in registry infrastructure to collect the necessary information to support health and social services planning.
  (e) Ensure that governments across the globe implement and use an internationally valid classification system for RDs (such as ICD11 or ORPHAcode) to effectively monitor and report on RDs.
6. Standardizing clinical guidelines
  (a) Streamline and standardize RD quality of care and treatment to ensure it is effective, efficient, and people-centered through the development and implementation of regionally and resource-relevant clinical guidelines for all types of RDs.
  (b) Ensure that regions and countries also have available resource-relevant clinical guidelines for all types of RDs. Countries and regions should ensure that global guidelines are adapted, contextualized, or updated to consider differences in available resources in different contexts.
7. Investing in capacity-building
  (a) Ensure that patients, through patient organizations, are kept informed and updated on changes in policies, regulations, and services that concern them.
  (b) Ensure countries and regions have an organized network of RD PAGs to share knowledge and experience, as well as to collectively engage in advocacy efforts.
  (c) Government should ensure the availability of mechanisms and spaces for a collaborative process with different stakeholders involved (providers, patients, key opinion leaders, payers, policymakers, academics, etc.), recognizing the role they can play in helping to develop and implement sound RD policies.
  (d) Ensure PAGs have the necessary resources to effectively shape discussions and influence policy decision-making.
8. Ensuring patients’ participation in decision-making
  (a) Empower patients and build the capacity to make their voices heard in the policy environment.
  (b) Ensure patients participate in decision-level conversations at the global, regional, and national levels, having in place mechanisms (at all levels) for their voices to be heard and counted.
  (c) Governments should have mechanisms and requirements for patients’ experiences to be considered during HTA processes.
9. Strengthening stewardship and accountability
  (a) Governments should leverage learnings and lessons from the experiences of other countries (best practices) to guide partnerships and decision-making.
  (b) Create/strengthen global and regional coalitions to channel the voices of patients and other relevant stakeholders to inform policymaking.
  (c) Foster initiatives that help countries access support from international organizations, other countries, etc.
  (d) Ensure mechanisms to assess progress towards policy commitments are available and implemented.
  (e) Acknowledge and create momentum for regulatory and policy change to ensure the regulatory system is up to date and HTA and Incremental Cost-Effectiveness Ratio (ICER) frameworks facilitate access to innovative RDs medicines that may not have a clear cost-benefit ratio.
  (f) Establish a regional coalition for a common approach to investigating and evaluating new types of diagnostics and treatments for RDs.
  (g) Provide support to manufacturers to help them understand and navigate the regulatory processes.
10. Building public awareness
  (a) Implement health promotion campaigns to raise awareness and combat stigma and discrimination against RD patients and their families.
  (b) Improve public–private partnerships and collaboration to leverage existing initiatives and expand their impact on the RD landscape.
  (c) Tackle the high economic impact of RDs by improving health services capabilities, including aspects of diagnosis, integration, and coordination of care, medical and clinical practitioners’ capacity, and care and clinical pathways.
11. Supporting the development of diagnostics
  (a) Support the development of, and access to, diagnostics and screening technologies (e.g., whole genome sequencing and NBS) to shed light on the true burden of disease.
  (b) Recognize and attend to the need to fund diagnostics (to drive natural history studies, for example) for incurable diseases (for now) to spur innovation.

## 5. Limitations

The methodology used to develop this paper (a review of literature and a process of discussion and validation by experts) contributed to creating a comprehensive narrative that considered a diversity of conditions, multiple dimensions, and myriad resources (from national to regional evidence) at the same time. Conveying all these dimensions had not been possible using a systematic review methodology. We do, however, acknowledge and understand that in consequence, the replicability of the study is limited. 

Furthermore, since data and evidence on RDs come from myriad resources, they cannot be seamlessly generalized across regions. This is also the case of policy-related evidence. While a summary of evidence on RD policies was included, extrapolation of this information must be performed carefully. This is especially true for lower-income countries, where RD policies are just developing and where intrinsic challenges persist in measuring outcomes.

Finally, while most references are peer-reviewed pieces, in some cases, to resolve gaps in evidence, gray literature was considered. In such cases, sources were appraised to ensure quality and credibility. 

## 6. Conclusions

RDs are often severe, chronic, and progressive, with high mortality associated with them. RDs have profound negative impacts on patients, their families, and societies, and RD patients often face many challenges to have their needs met. RDs are only recently and progressively becoming a policy priority for the UN and WHO, extending an invitation to increase health policies and initiatives at the regional and country levels. The SDGs share the vision of a world in which no one is left behind and in which health equity is promoted. Responding to and resolving the needs of the RD community is paramount to achieving this vision, a world where in fact no one is left behind. 

While important steps have been taken to raise awareness of RDs and encourage the development of international and national frameworks and policies for people living with RDs, many challenges persist. The journeys of RD patients to have their healthcare needs addressed and their diseases managed include many barriers, leading to the worst consequences on patients’ and their caregivers’ lives. From a wider scope, RDs have profound economic and societal costs. 

In this document, we discussed these dimensions and key takeaways both from a global perspective and considering the experiences of each region. We also discussed the RD policies landscape, roughly capturing what kind of measures, policies, and regulations are in place in different regions, drawing lessons that the international community can learn from. Nonetheless, it is important to acknowledge that interpretation and extrapolation of this information must be performed carefully. This is especially true for lower-income countries, where RD policies are just developing and where intrinsic challenges persist. 

Finally, as a result of our analysis, we provide a broad range of recommendations that countries and international decision makers can use to prioritize and identify strategies needed to address the challenges faced by the RD community. The applicability and feasibility of these recommendations should be assessed according to the context and local resources and needs.

## Data Availability

Not applicable.
